# Bioprocess and biotecnology: effect of xylanase from *Aspergillus niger* and *Aspergillus flavus* on pulp biobleaching and enzyme production using agroindustrial residues as substract

**DOI:** 10.1186/2193-1801-2-380

**Published:** 2013-08-13

**Authors:** Nelciele Cavalieri de Alencar Guimaraes, Michele Sorgatto, Simone de Carvalho Peixoto-Nogueira, Jorge Henrique Almeida Betini, Fabiana Fonseca Zanoelo, Maria Rita Marques, Maria de Lourdes Teixeira de Moraes Polizeli, Giovana C Giannesi

**Affiliations:** Laboratory of Biochemistry, CCBS - Universidade Federal de Mato Grosso do Sul/UFMS, Av Costa e Silva s/n°, Campo Grande, MS 79070-900 Brazil; Department of Biology, Faculdade de Filosofia, Ciências e Letras de Ribeirão Preto, Universidade de São Paulo, Av do Café s/n°, Ribeirão Preto, SP 14040-901 Brazil

**Keywords:** *Aspergillus niger*, *Aspergillus flavus*, Agroindustrial residues, Wheat bran, Biobleaching

## Abstract

This study compares two xylanases produced by filamentous fungi such as *A. niger* and *A. flavus* using agroindustrial residues as substract and evaluated the effect of these enzymes on cellulose pulp biobleaching process. Wheat bran was the best carbon source for xylanase production by *A. niger* and *A. flavus*. The production of xylanase was 18 and 21% higher on wheat bran when we compare the xylanase production with xylan. At 50°C, the xylanase of *A. niger* retained over 85% activity with 2 h of incubation, and *A. flavus* had a half-life of more than 75 minutes. At 55°C, the xylanase produced by *A. niger* showed more stable than from *A. flavus* showing a half-life of more than 45 minutes. The xylanase activity of *A. niger* and *A. flavus* were somehow protected in the presence of glycerol 5% when compared to the control (without additives). On the biobleaching assay it was observed that the xylanase from *A. flavus* was more effective in comparison to *A. niger*. The kappa efficiency corresponded to 36.32 and 25.93, respectively. That is important to emphasize that the cellulase activity was either analyzed and significant levels were not detected, which explain why the viscosity was not significantly modified.

## Introduction

Annually, large quantities of lignocellulosic wastes (agricultural and agroindustrial residues, like wheat bran, sugarcane bagasse, soybean, rice straw, corncob and orange peel) are generated through industrial processes such as breweries, paper-pulp, textile and timber industries. And their disposal is becoming a problem regarding space, causing environmental pollution because most of the wastes are disposed by burning. However, the plant biomass regarded as “wastes” are biodegradable and represent an inexpensive alternative source for microbial growth or enzymes production (Sepahy et al., [2011]; Facchini et al., [2011]; Okafor et al., [2007]).

In the last decades, an increasing number of studies aimed to develop environmentally clean and non-toxic methods for industrial processes (Betini et al., [2009]; Peixoto-Nogueira et al., [2009]). The possibility of using a variety of agricultural residues is an attractive practice for the production of plant cell wall degrading hydrolytic enzymes, especially xylanase and cellulase, because this system could simulate the natural environment (Facchini et al., [2011, 2012]).

The xylanase production has been reported for bacteria (Sepahy et al., [2011]), actinomycetes (Garg et al., [2011]) and fungi (Das et al., [2013]; Sorgatto et al., [2012]; Facchini et al., [2011, 2012]). Filamentous fungi which produce xylanases are attracting greater attention than bacteria and yeast because they are particularly interesting from an industrial point of view, due to the fact they secrete much higher xylanolytic enzymes into the medium (Okafor et al., [2007]; Polizeli et al., [2005]). Among the filamentous fungi employed to produce xylanase, the *Aspergillus* genus is one of the most explored (Sandrim et al., [2005]; Betini et al., [2009]; Peixoto-Nogueira et al., [2009]; Michelin et al., [2010]). To date, however, there have been few reports of xylanases produced by *Aspergillus flavus*.

Xylanases produced by microorganisms has attracted a great deal of attention during the past few decades because of its potential biotechnological applications in various industries including food, feed, fuel, textile, and paper and pulp industries and in waste treatment (Yeasmin et al., [2011]; Facchini et al., [2011]; Michelin et al., [2010]; Jiang et al., [2010]).

An increasing awareness on environmental pollution has enforced the pulp and paper industries to strive for an alternate greener technology which will replace the use of harsh chemicals in their processes with microbial enzymes. The application of biocatalysts not only makes the process less toxic but also decreases costs associated with the production and consumption of resources (water, electricity, fuels) (Birijlall et al., [2011]).

At present, bleaching process for kraft pulp uses large amounts of chlorine-based chemicals and sodium hydrosulfite, which are toxic, mutagenic, and persistent; they also cause numerous harmful disturbances in biological systems (Yeasmin et al., [2011]). The enzymatic step in the process of cellulose pulp bleaching contributes to reduce the use of chlorine-containing reagents (Peixoto-Nogueira et al., [2009]). Xylanases aid in catalyze the hydrolysis of glycosidic bonds in the xylan backbone, reducing the degree of polymerization of the substrate, enhancing the brightness of pulp (facilitating the chemical extraction of lignin from pulp in subsequent alkaline extraction) and diminish impurities. Cellulose-free, alkali and thermo-stable microbial xylanases are mostly ideal for biopulping and bleaching processes (Birijlall et al., [2011]; Yeasmin et al., [2011]; Peixoto-Nogueira et al., [2009]).

This paper shows an important comparing of the xylanase production by *A. niger* and *A. flavus* using agroindustrial residues as carbon sources and tested their suitability for cellulose pulp biobleaching. These fungi produced xylanases with special characteristics, such as high pH stability and high optimum temperature, in comparison to others reported in the literature (Facchini et al., [2011]; Sepahy et al., [2011]). And the xylanase activity from *A. niger* was the most thermostable of three enzyme samples (*A. niger*, *A. flavus* and *A. japonicus* var *aculeatus*, studied before in our laboratory).

## Materials and methods

### Microorganisms

*Aspergillus niger* and *Aspergillus flavus* strains were isolated by us from soil samples, identified in the Federal University of Pernambuco – UFPE (PE, Brazil) and deposited in our laboratory fungi collection. Stock cultures were propagated at 30°C on slants of solid potato dextrose agar (PDA) media and stored at 4°C.

### Xylanase production and enzyme extraction

Spores were inoculated into 125 ml Erlenmeyer flasks containing 25 ml media (Rizzatti et al., [2001]) using 1% (w/v) of the desired carbon sources (xylan or agroindustrial residues such as wheat bran, rice bran, sugarcane bagasse or corncob). The cultures were incubated under orbital agitation (110 rpm) condition, at 30°C, during five (*A. niger*) or two days (*A. flavus*), already standardized in our laboratory. The media was subsequently vacuum-filtered using filter paper (Whatman n° 1) and the crude filtrate was used for the study of the extracellular enzyme.

### Enzymatic assays and protein determination

The reaction mixture consisted in 500 μl of citrate-phosphate buffer (McIlvaine, [1921]) pH 5.0 for both fungi containing 1% (w/v) of xylan (birchwood), and 500 μl of enzymatic extract appropriately diluted. Identical conditions of assay were employed for cellulase determination, using as substrate 1% (w/v) carboxymethyl-cellulose. The samples were incubated at 60°C to determine xylanase and cellulase activity. The amount of reducing sugar released was determined using the 3,5-dinitrosalicylic acid (DNS) method (Miller, [1959]), employing xylose (Sigma) (xylanase) and glucose (cellulase) as the standards. One unit of enzyme activity was defined as the amount of enzyme which releases 1 μmol of reducing sugar per minute under assay condition. Specific activities were expressed as U/mg of protein. Protein concentrations were determined by the Lowry et al. ([1951]), using bovine serum albumin (BSA) as the standard.

### Effect of temperature and pH on enzyme activity

The effects of temperature and pH on xylanase activity were analyzed using crude extracts from *A. niger* and *A. flavus*. The optimum temperature and pH for *A. niger* and *A. flavus* were 60°C and 5.0, respectively (results not shown). The thermal stability was determined with enzymes incubated between 45 and 55°C for different periods (5 to 120 min). The influence of protectors on xylanolytic activities of *A. niger* and *A. flavus* was tested at 55 and 50°C, respectively, during 120 minutes in the presence of 5% glycerol or polyethyleneglycol. The pH stability was analyzed using McIlvaine buffer in the pH range 3.0 - 8.0 for 1 hour.

### Biobleaching

The amount of enzyme used from *A. niger* or *A. flavus* for this treatment was 10 units of enzyme per gram of dried cellulose pulp from *Eucalyptus grandis*. All calculations and procedures were determined according to the standard methods of Technical Association of the Pulp and Paper Industry (TAPPI test methods [1996]). The consistency was determined on a percent dry weight basis. The volume of enzyme or distilled water was added until it reached a 10% pulp consistency. Crude xylanase extracts from *A. niger* and *A. flavus* were added to the treated pulp and the control was prepared by adding distilled water instead of enzyme. The samples were incubated inside sealed polyethylene bags at 55°C for 2 hours and after that, the treated cellulose pulps were filtered on a Büchner funnel, rinsed with 200 ml of distilled water and used for determination of kappa number and viscosity. The filtrate was used to analyze the liberation of aromatic compounds monitored by absorbance values at 237 and 465 nm.

### Reproducibility of results

All results are expressed as the means of at least three independent experiments.

## Results and discussion

### Xylanase production using different agroindustrial residues and substrates

When different carbon sources were tested (Table 1) it was observed that the wheat bran 1% was the best carbon source for xylanase production by *A. niger* (12.76 U/mg of protein), followed by fine sugarcane bagasse 0.5% (10.64 U/mg of protein) and corncob 1% (10.00 U/mg of protein). The production of xylanase was 18 (oat spelt xylan 1%) and 21% (birchwood xylan 1%) higher on wheat bran 1% when we compare the xylanase production with xylan (10.43 and 10.10 U/mg of protein, respectively), which means that it can substitute the xylan efficiently. In studies with *A. fumigatus* RP04*,* the production of xylanase was only between 5 and 6% higher on agroindustrial residues (powdered corncob, wheat bran and crushed corncob) when compared with media containing birchwood xylan as carbon source (Peixoto-Nogueira et al., [2009]). Although the xylanase production was elevated in birchwood xylan 0.5% (15.72 U/mg of protein), the utilization of an alternative carbon source, like wheat bran or sugarcane bagasse, still is viable due to its low cost for enzyme production, reducing these wastes in the environment. For *A. flavus*, the xylanase production was better induced by wheat bran 1 and 0.5% (8.03 and 8.70 U/mg of protein, respectively), followed by fine sugarcane bagasse 1% (7.68 U/mg of protein). The fine sugarcane bagasse was autoclaved, dried and ground. The other substrates tested did not presented significant results for both fungi.

**Table 1:** **Effect of different carbon sources on xylanase production**

	***Aspergillus niger***			***Aspergillus flavus***		
Carbon sources	Activity	Protein	Specific activity	Activity	Protein	Specific activity
	(U/ml)	(mg/ml)	(U/mg of protein)	(U/ml)	(mg/ml)	(U/mg of protein)
Glucose 1.0%	0.04	0.37	0.11 (±0.09)	0.18	0.79	0.23 (±0.03)
Rice bran 1.0%	2.77	0.67	4.13 (±0.45)	0.11	0.97	0.11 (±0.01)
Rice bran 0.5%	1.13	0.54	2.09 (±0.37)	0.09	0.97	0.09 (±0.01)
Rice straw 1.0%	0.97	0.45	2.16 (±0.10)	2.92	0.91	3.21 (±0.12)
Rice straw 0.5%	0.48	0.33	1.46 (±0.39)	1.87	0.71	2.63 (±0.02)
Coarse sugarcane bagasse 1.0%	5.49	0.72	7.63 (±0.45)	2.64	0.75	3.52 (±0.27)
Coarse sugarcane bagasse 0.5%	2.78	0.61	4.56 (±0.43)	0.63	0.66	0.96 (±0.14)
Fine sugarcane bagasse 1.0%	5.96	0.78	7.64 (±0.44)	5.53	0.72	7.68 (±0.29)
Fine sugarcane bagasse 0.5%	5.32	0.50	10.64 (±0.45)	2.33	0.97	2.40 (±0.09)
Corncob 1.0%	8.00	0.80	10.00 (±0.31)	3.22	0.86	3.74 (±0.07)
Corncob 0.5%	5.84	0.64	9.13 (±0.24)	1.65	0.62	2.67 (±0.44)
Wheat bran 1.0%	8.42	0.66	12.76 (±0.24)	11.57	1.44	8.03 (±0.48)
Wheat bran 0.5%	6.23	0.69	9.03 (±0.27)	10.26	1.18	8.70 (±0.03)
Xylan (oat spelt) 1.0%	6.05	0.58	10.43 (±0.31)	11.71	0.87	13.46 (±0.38)
Xylan (oat spelt) 0.5%	6.61	0.59	11.20 (±0.24)	10.38	0.87	11.94 (±0.41)
Xylan (birchwood) 1.0%	7.27	0.72	10.10 (±0.13)	11.22	0.55	20.40 (±0.21)
Xylan (birchwood) 0.5%	9.12	0.58	15.72 (±0.05)	11.64	0.74	15.72 (±0.13)
Corncob 0.5% + Fine sugarcane bagasse 0.5%	9.25	0.52	17.79 (±0.50)	3.59	0.76	4.72 (±0.02)
Wheat bran 0.5% + Fine sugarcane bagasse 0.5%	9.90	0.52	19.04 (±0.37)	10.77	1.23	8.75 (±0.37)
Wheat bran 0.5% + Corncob 0.5%	10.50	0.52	20.19 (±0.36)	11.92	1.07	11.14 (±0.01)

*A. niger* and *A. flavus* were grown on SR liquid media (Rizzatti et al., [2001]) and different carbon sources, which were incubated for five and two days, respectively, at 30°C under orbital agitation (110 rpm) condition. The assays were performed at 60°C and McIlvaine buffer pH 5.0.

The universal suitability of wheat bran as substrate is because of its cell-wall polysaccharides that contain 40% xylan, and it does not aggregate, even under high moisture conditions, providing a large surface area (Dhillon et al., [2011]; Betini et al., [2009]). It is why in literature can be found a lot of works of xylanase production using wheat bran, for several Aspergillus species like, *Aspergillus terreus* (Sorgatto et al., [2012]); *Aspergillus niger* BCC14405 (Khonzue et al., [2011]); *Aspergillus niger* BC-1 (Dhillon et al., [2011]); *Aspergillus terricola* Marchal (Michelin et al., [2010]); *Aspergillus foetidus* MTCC 4898 (Chapla et al., [2010]); *Aspergillus niger* (Farinas et al., [2010]); *Aspergillus niger* DFR-5 (Pal and Khanum, [2010]); *Aspergillus niveus* RP05 (Peixoto-Nogueira et al., [2009]); *Aspergillus niger*, *Aspergillus niveus* and *Aspergillus ochraceus* (Betini et al., [2009]) and *Aspergillus ficuum* AF-98 (Fengxia et al., [2008]). Xylanase production on corncob and sugarcane bagasse was also reported for *Aspergillus terreus* (Sorgatto et al., [2012]), *Chaetomium* sp. CQ31 (Jiang et al., [2010]), *Aspergillus foetidus* MTCC 4898 (Chapla et al., [2010]), *Aspergillus terreus* (Lakshmi et al., [2009]), *Thermomyces lanuginosus* MC 134 (Kumar et al., [2009]) and *Aspergillus niger* ANL 301 (Okafor et al., [2007]).

In the tests of influence of mixtures (1:1) of substrates, the xylanase production by *A. niger* was 22% higher on the mixture of wheat bran and corncob when compared to media containing xylan as carbon source. When we compare the xylanase production by *A. flavus* in this mixture, it was almost 45% lower than from *A. niger*. And comparing the xylanase production between the used fungi in the other mixtures, *A. flavus* presented a very lower production than *A. niger.*

In literature, different combinations of agroindustrial residues as carbon sources for xylanase production, has been reported for several fungi (Khonzue et al., [2011]; Facchini et al., [2011]; Dhillon et al., [2011]; Pal and Khanum, [2010]; Betini et al., [2009]; Peixoto-Nogueira et al., [2009]). *Aspergillus niger* DFR-5 had his highest xylanase production in media containing a mixture of wheat bran and soybean powder in a ratio 7:3 (Pal and Khanum, [2010]), while *Aspergillus niger* BC-1 mixed with *T. reesei* Rut C-30 (ATCC 56765) had the highest activity with rice straw and wheat bran (3:2) (Dhillon et al., [2011]). Facchini et al. ([2011]) found that a mixture of soybean meal and crushed corncob increased xylanolytic activity by only 8.5%. And in work using the fungus *A. niveus* and some agroindustrial residues, wheat bran mixed with corncob not improved the production of xylanase, but *A. niger* and *A. ochraceus* had an increase of approximately 20% (Betini et al., [2009]).

For the enzyme characterization and the biobleaching study, there were separated the filtrates that more produced enzymes. And the cellulase activity of theses filtrates was determined and did not detect significant levels (data not shown).

### Effect of temperature and pH on xylanase stability

**Figure 1:**
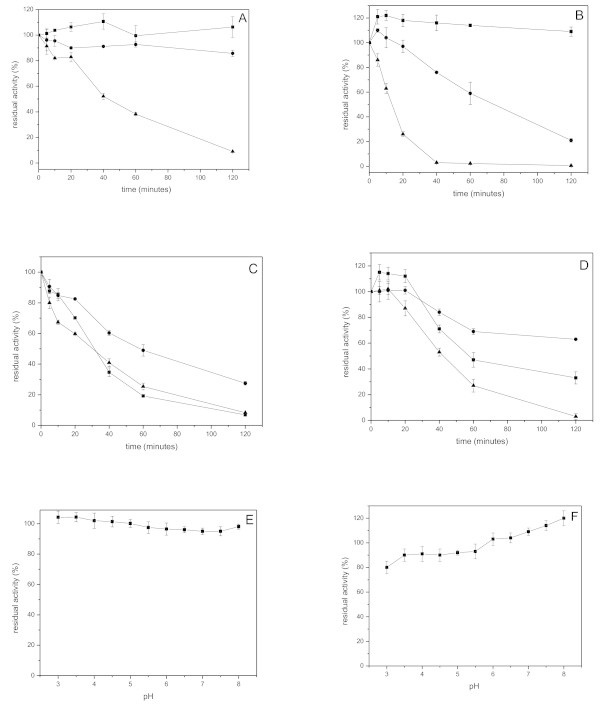
**Characterization of the xylanases from*A. niger*and*A. flavus.*** Thermostability of the xylanase of *A. niger***(A)** and *A. flavus***(B)** were determined using McIlvaine buffer pH 5.0 at 60°C after incubating the enzymes on temperatures of 45 (■), 50 (●) and 55°C (▲). Thermostability of the xylanases at 55 (*A. niger*) **(C)** and 50°C (*A. flavus*) **(D)**, without additives (■), and with 5% glycerol (●) and polyethyleneglycol (▲). pH stability of *A. niger***(E)** and *A. flavus***(F)** were determined incubating both enzymes at different pHs (3.0 - 8.0) at 0°C, during 1 hour and after the residual activities were determined at 60°C, pH 5.0.

The xylanases produced by *A. niger* and *A. flavus* remained totally stable at 45°C. At 50°C both xylanases were stable for the first twenty minutes, where the xylanase of *A. niger* (Figure 1A) still retained 85.72% activity with 2 hours of incubation, and *A. flavus* (Figure 1B) had a half-life of more than 75 minutes. At 55°C, the xylanase produced by *A. niger* showed more stable than from *A. flavus* showing a half-life of approximately 45 minutes. In studies with *A. casielus* (Kronbauer et al., [2007]), *A. phoenicis* (Chipeta et al., [2005]) and *A. giganteus* (Coelho and Carmona, [2003]) the xylanases had half-life of only 50, 25 and 13 minutes at 50°C, respectively. The xylanase produced by *A. terreus* FSS129 had a residual activity of only 70% approximately with 2 hours of incubation at 50°C, and just retained 25.4% activity at 55°C after 1 hour exposure, while our *A. niger* still retained 38.2% activity (Bakri et al., [2010]). And at 55°C the xylanase of *A. casielus* had a half-life of only 17 minutes (Kronbauer et al., [2007]).

The influence of glycerol and polyethyleneglycol as enzyme protectors was also tested (Figure 1C,D). The addition of 5% glycerol somehow protected significantly the xylanase produced by *A. niger* from thermal inactivation at 55°C during all the incubation period. The xylanase activity increased 20.66% with 2 hours of incubation when compared to the control (without additives), and it was observed a half-life of approximately 32 minutes to the control and 60 minutes with glycerol (Figure 1C). And the xylanase activity of *A. flavus* increased 30% with 2 hours of incubation at 50°C in the presence of glycerol, which was not sufficient time to get the enzyme half-life (Figure 1D). The polyethyleneglycol was not effective as protector for both enzymes.

Regarding pH stability, it was verified that the xylanase from *A. niger* (Figure 1E) was totally stable at all the pHs tested, retaining more than 95% activity during 1 hour, while the xylanase from *A. flavus* (Figure 1F) had a small decrease of 10% at pH between 3.0 – 5.5, maintaining stable (100%) at higher pHs. The stability of the xylanases of *A. niger* and *A. flavus* under alkaline pH is very interesting when we remember that the biobleaching process with xylanases is done under alkaline conditions. Michelin et al. ([2010]) related a xylanase produced by *A. terricola* Marchal and *A. ochraceus* that retained more than 70% of its original activity in a pH range of 2.5 – 8.0 for 1 hour. The xylanase produced by *A. fumigatus* was stable just in pH from 6.0 to 8.0, while the xylanase from *A. niveus* was more stable from 4.5 to 6.0 (Peixoto-Nogueira et al., [2009]). The xylanases of *A. niger*, *A. niveus* and *A. ochraceus* were stable in the range of pH 2 – 7 retaining more than 70 – 80% activity (Betini et al., [2009]).

### Assays of cellulose biobleaching using xylanase

To analyze the xylanases efficiency for cellulose pulp biobleaching assay, the cellulose pulp was clarified by *A. flavus* or *A. niger* crude extract. The procedures occurred at 55°C for 2 h and it was observed that the xylanase crude extract from *A. flavus* was more effective in comparison to *A. niger*. The kappa number reduced 5.07 and 3.62 points, respectively, which corresponds to 36.32 and 25.93 kappa efficiency, respectively (Table 2).

**Table 2:** **Properties of pulp treated with xylanases produced by*A. niger*and*A. flavus***

Parameters	Control	***A. niger***	***A. flavus***
Kappa number	13.96	10.34	8.89
Kappa efficiency (%)	-	25.93	36.32
CST (%)	21.70	21.30	21.60
A_237 nm_	-	0.093	0.120
A_465 nm_	-	0.056	0.062

Biobleaching of bagasse pulp with 10 U xylanase g^-1^ of pulp in 0.5 M sodium citrate buffer (pH 6.5) at 55°C for 2 h.

This efficiency can be also proved by cromophores liberation, which was redden at 237 and 465 nm and again was bigger when the cellulose pulp was clarified by *A. flavus* crude extract. That is important to emphasize that the cellulase activity was either analyzed and significant levels were not detected (data not shown), which explain why the viscosity was not significantly modified.

Comparing the used fungi that is clear that the xylanase from *A. flavus* is more effective in comparison to xylanase from *A. niger*. These advantages can also be seen when you compare the obtained results for *A. flavus* and *A. niger* to some data from the literature. The xylanase of *A. caespitosus* (10 U/g dry pulp/ 2 hours) reduced kappa number only in 12.6% (xyl II) and 1.7% (xyl I) (Sandrim et al., [2005]), while the *A. flavus* and *A. niger* xylanases kappa efficiency corresponded to 36.32 and 25.93%, respectively. Medeiros et al. ([2007]) reported xylanases from *T. longibrachiatum*, *P. corylophilum* and *A. niger* that reduced only 1.1, 0.5 and 0.6 points the kappa number with 5 U/g dry pulp/ 4 hours; and *Aspergillus fumigatus* ABK9 reduced only 0.7, 1.2, 2.7, 3.3 and 4 points the kappa number, using 20, 40, 60, 80 and 100 U/g dry pulp/ 6 hours, respectively (Das et al., [2013]). And Kumar et al. ([2009]) reported a xylanase (50 U/g dry pulp/ 3 hours) from *T. lanuginosus* MC 134 that reduced only 3.2 points the kappa number.

## Conclusions

*A. niger* and *A. flavus* showed that are good xylanase producers and these enzymes can be obtained using alternative carbon sources such as wheat bran, corncob and fine sugarcane bagasse, that is important to emphasize that the xylanase production increased when a mix of residues was used, showing a better production than on media supplemented only by xylan, proving that they can substitute the specific substrate and consequently reduce the enzyme production cost, since the commercial xylan is extremely expensive for widespread use. The xylanase from A. *niger* showed to be stable for temperature and pH, while the *A. flavus* xylanase was not so stable for temperature but showed excellent stability at high pH (6–8), characteristics that are very important for the biobleaching of cellulose pulp process. On biobleaching assay the xylanases from *A. flavus* were more effective in comparison to xylanases from *A. niger*.
